# Nonlinear Dynamic Analysis of Vowels in Cleft Palate Children with or without Hypernasality

**DOI:** 10.1155/2012/739523

**Published:** 2011-12-06

**Authors:** Katsuaki Mishima, Hiroyuki Nakano, Tatsushi Matsumura, Norifumi Moritani, Seiji Iida, Yoshiya Ueyama

**Affiliations:** ^1^The Department of Oral and Maxillofacial Surgery, Graduate School of Medicine, Yamaguchi University, Minami-kogushi 1-1-1, Ube City, Yamaguchi 7558505, Japan; ^2^The Department of Oral and Maxillofacial Reconstructive Surgery, Graduate School of Medicine, Dentistry and Pharmaceutical Sciences, Okayama University, Shikata-cho 2-5-1, Okayama City, Okayama 7008525, Japan

## Abstract

*Objectives*. To clarify the difference between Lyapunov exponents (LEs) for cleft palate (CP) patients with hypernasality versus without hypernasality and to investigate the relationship between their LEs and nasalance scores (NSs). *Material and Methods*. Six CP patients with severe hypernasality (mean age 9.2 years) and six CP patients without hypernasality (mean age 8.0 years) were enrolled. Five Japanese vowels were recorded at 44.1 KHz, and the NSs were measured simultaneously. The mean first LE (mLE_1_) from all one-second intervals was computed. *Results*. The mLE_1_ for /o/ in patients with hypernasality was significantly higher than that in patients without hypernasality. The correlation coefficients between the mLE_1_ and NS for all vowels were not statistically different. *Conclusion*. The voice signal of /o/ for the patients with hypernasality was more instable than in those without hypernasality. The chaotic phenomenon was independent of nasal resonance in CP voice.

## 1. Introduction

Patients with cleft palate often exhibit nasality, which is a distinctive feature and an important target in speech therapy and rehabilitation. To evaluate velopharyngeal function, the aerodynamic and acoustic aspects of nasalization have been studied. An aerodynamic exam can diagnose the degree of velopharyngeal closure [1, 2], and acoustic measurements can categorize velopharyngeal insufficiency [3, 4]. The abnormal resonance generated by velopharyngeal insufficiency can be evaluated quantitatively using a nasometer [5].

On the other hand, the voice and speech of patients with cleft palates have been studied using many techniques including spectral analysis, perturbation analysis, and formant analysis. Zajac and Linville [6] and Lewis et al. [7] reported that cleft palate speakers have larger frequency perturbations (jitter) than normal controls. However, the methods used to calculate perturbations, jitter, and shimmer are only reliable for nearly periodic voice signals and cannot reliably analyze strongly aperiodic signals [8]. Recently, nonlinear dynamic methods have enabled the quantification of aperiodic and chaotic phenomena [9–11].

In our previous paper, we reported that the Lyapunov exponents (LEs) of the vowels /a/, /e/ and /o/ for adult cleft palate patients are higher than those for normal resonance adults and that there were no correlation coefficients between LEs and nasalance scores (NSs) [12]. These results suggested that vocal fold vibration may be less stable in adult cleft palate patients than in normal resonance subjects and that the LE may be a parameter independent of resonance. Subsequently, we investigated the nonlinear dynamic characteristics of cleft palate speech and voice. In the present paper, the purpose was to clarify the difference between the LEs for cleft palate patients with hypernasality versus without hypernasality and to investigate the relationship between their LEs and NSs. 

## 2. Materials and Methods

### 2.1. Patients

Six repaired cleft palate patients with severe hypernasality (mean age 9.2 years; range 6 to 13, 2 boys and 4 girls) and six repaired cleft palate patients without hypernasality (mean age 8.0 years; range 6 to 13, 4 boys and 2 girls) were enrolled. The presence of hypernasality was perceptually judged by two speech therapists from Okayama University Hospital. The present study, which was approved by the Okayama University Institutional Ethical Board, was carried out after obtaining informed consents from the parents of all participants.

### 2.2. Methods

The voices were recorded through a microphone (Shure BG1.1, Niles, Ill) on a portable solid-state recorder (Marantz PMD 640, Itasca, Ill) with a nasometer II headset (model 6400, Kay Elemetrics Corp., Lincoln Park, NJ) in a quiet room designated for speech therapy in the Okayama University Dental Hospital. The nasalance scores (NSs) were measured simultaneously. The voice samples were recorded on the compact flush medium of the recorder at a sampling rate of 44.1 KHz, at 16 bits, in a *.wav file format. The Japanese vowels /a/; [a], /i/; [i], /u/; [*ɯ*], /e/; [e], and /o/; [o] were used as voice samples. Each vowel was naturally phonated during approximately one-second three times.

The voice data were processed on a personal computer (NEC mate MA30Y, Tokyo) with a modified Chaos Analyzing Program (ver. 1.0.4, CCI Corporation, Fukuoka), which used the algorithm from Sano and Sawada [13]. The first Lyapunov exponent (LE_1_) was computed for each one second interval, while the interval was being shifted by 100 msec. The mean first Lyapunov exponents for all intervals (mLE_1_) were then calculated.

The delay time was determined as follows. The first zero-crossing points of autocorrelation were calculated for each vowel. As a result, the delay time was estimated at 15, 32, 27, 22, and 21 points (1 point = 1/44.1 msec.) for the vowels /a/, /i/, /u/, /e/, and /o/, respectively (Table 1). The embedding dimension was determined as follows. The fractal dimensions were computed using the Grassberger-Procaccia algorithm [14], and convergent diagrams, in which the embedding dimensions were assumed, were then constructed for each vowel. Thus, the embedding dimensions were estimated at 5 for all vowels (Table 1).

The differences of the first zero-crossing points of autocorrelation, estimated the embedding dimensions, and the mLE_1_s between the two groups with versus without hypernasality were analyzed statistically using the Mann-Whitney *U* test. The correlation coefficients between the mLE_1_ and NS were also calculated for each vowel. The statistical package SPSS (ver.16.0) was utilized, and differences with *P* values of less than 0.05 were considered to be statistically significant. 

## 3. Results

There were no significant differences between the first zero-crossing points and the estimated embedding dimensions of those patients with or without hypernasality for all vowels (Table 1). The mLE_1_ for /o/ in the patients with hypernasality was significantly higher than in patients without hypernasality (*P* = 0.015) (Table 2). The NSs for /i/, /u/, /e/, and /o/ in the patients with hypernasality were significantly higher than in patients without hypernasality (Table 3). The correlation coefficients between the mLE_1_ and NS for all vowels were not statistically different (Table 4).

## 4. Discussion

Although nasality can be evaluated using a spectral analysis of speech signals, voice acoustic measures of nasality are not universally used in clinical or empirical work because of ambiguity in the literature regarding the appropriate acoustic methodology, the amount of labor involved as compared with the Nasometer, and so forth [15]. However, Vogel et al. [15] demonstrated the potential for the wider application of acoustic investigation into nasality.

Several authors have described laryngeal disorders, including organic and functional disorders, in cleft palate speakers [16, 17]. Zajac and Linville [6] and Lewis et al. [7] reported that cleft palate speakers have higher frequency perturbations (jitter) than normal controls. Nicollas et al. [18] demonstrated that neither jitters nor shimmers significantly differed with age or gender. Van Lierde et al. [19] reported a multiparameter approach to vocal quality but stated that the nature of the vocal quality and the voice range measurement differences cannot be explained from their study. Therefore, we concluded that future studies on the voice of cleft palate subjects using nonlinear analysis may be beneficial in gaining further insight into the mechanics of phonation.

To our knowledge, there have been no reports on the application of nonlinear dynamic analysis to cleft palate speech. Our previous study demonstrated that the mLE_1_ for /a/, in both males and females with CP, is significantly higher than in normal resonance individuals and that the mLE_1_ for /e/ in males with CP and for /o/ in females with CP are significantly higher than in normal resonance individuals [12]. Since the mLE1 is a measure of the instability of the voice signal, these results suggest that the vocal fold vibration is less stable in CP speakers than in normal resonance subjects. In addition, the correlation coefficients between the mLE_1_ and NS for all vowels were not statistically different in both normal and CP subjects. Therefore, the Lyapunov exponents may be independent of resonance [12]. Subsequently, the nonlinear dynamic characteristics were investigated in the present study. The mLE_1_ for /o/ in the patients with hypernasality was significantly higher than in patients without hypernasality; in other words, the voice signal of /o/ for the patients with hypernasality was more instable than in those without hypernasality. This may contribute to the instability of the vocal fold. On the other hand, the correlation coefficients between the mLE_1_ and NS for all vowels were not statistically different in patients both with versus without hypernasality. This supported the independence of chaotic phenomenon and nasal resonance in cleft palate speech and voice, which was demonstrated in our previous paper [12].

Nicollas et al. [18] reported that the large LE seems to decrease with age from their studies of children between 6 and 12 years of age. It was also suggested that the large LE is lower in boys than in girls overall but varies for each age [18]. In our present study, the boys and girls were not separated because of the small sample size. A further investigation is necessary in this respect.

## 5. Conclusions

The voice signal of /o/ for the patients with hypernasality was more instable than in those without hypernasality. The chaotic phenomenon was independent of nasal resonance in cleft palate speech and voice.

## Figures and Tables

**Table 1:** First zero-crossing points of autocorrelation and estimated embedding dimensions.

	*n*	/a/	/i/	/u/	/e/	/o/
Hypernasality (+)	6	15.2 ± 3.4	31.5 ± 5.8	25.8 ± 3.4	20.7 ± 3.6	22.7 ± 10.7
		4.4 ± 0.6	4.4 ± 0.9	4.6 ± 1.1	4.4 ± 0.6	4.8 ± 1.3
Hypernasality (−)	6	14.8 ± 5.6	32.0 ± 8.4	28.5 ± 9.0	22.5 ± 5.9	19.7 ± 4.4
		6.0 ± 1.2	5.8 ± 1.3	5.2 ± 1.6	5.8 ± 1.3	6.2 ± 1.8
*P* value		0.429	1.000	0.931	0.931	0.662
		0.063	0.190	0.286	0.063	0.111
Total	12	15.0 ± 4.4	31.8 ± 5.4	27.2 ± 6.6	21.6 ± 4.7	21.2 ± 8.0
		5.2 ± 1.3	5.1 ± 1.4	4.8 ± 1.4	5.1 ± 1.3	5.4 ± 1.7

Upper column: first zero-crossing points of autocorrelation (unit point; 1 point = 1/44.1 msec.),

lower column: estimated embedding dimensions.

**Table 2:** Lyapunov exponents (mLE_1_).

	*n*	/a/	/i/	/u/	/e/	/o/
Hypernasality (+)	6	2334.8 ± 796.7	787.2 ± 137.5	900.3 ± 243.2	1170.5 ± 236.0	1370.5 ± 280.9
Hypernasality (−)	6	1504.3 ± 557.2	703.3 ± 241.4	796.0 ± 322.5	1102.2 ± 279.8	839.3 ± 286.4
*P *value		0.093	0.310	0.589	0.699	0.015

mLE_1_; mean of the first Lyapunov exponents.

**Table 3:** Nasalance scores (NSs).

	*n*	/a/	/i/	/u/	/e/	/o/
Hypernasality (+)	6	18.3 ± 12.5	38.2 ± 12.0	23.8 ± 8.9	23.5 ± 10.5	20.7 ± 8.5
Hypernasality (−)	6	11.2 ± 4.6	18.3 ± 9.9	11.3 ± 6.9	12.2 ± 6.2	11.0 ± 5.4
*P *value		0.240	0.026	0.026	0.026	0.041

**Table 4:** Correlation coefficients between the mLE_1_ (mean of the first Lyapunov exponents) and NS (nasalance score).

	mLE_1_ of /a/	mLE_1_ of /i/	mLE_1_ of /u/	mLE_1_ of /e/	mLE_1_of /o/
NS of /a/	0.177				
	0.582				
NS of /i/		0.430			
		0.163			
NS of /u/			0.420		
			0.174		
NS of /e/				0.131	
				0.685	
NS of /o/					0.424
					0.170

Upper column: correlation coefficients, lower column: *P* value.
